# Differentiating electrocardiographic indications of massive and submassive pulmonary embolism: A cross‐sectional study in Southern Iran from 2015 to 2020

**DOI:** 10.1002/clc.24252

**Published:** 2024-03-11

**Authors:** Zahra Bahreini, Maliheh Kamali, Fatemeh Kheshty, Hamed Bazrafshan Drissi, Shahrokh Sadeghi Boogar, Mehdi Bazrafshan

**Affiliations:** ^1^ Cardiovascular Research Center Shiraz University of Medical Sciences Shiraz Iran; ^2^ Department of Internal Medicine Shiraz University of Medical Sciences Shiraz Iran

**Keywords:** electrocardiogram, electrocardiography, high‐risk pulmonary embolism, massive pulmonary embolism, pulmonary embolism

## Abstract

**Background:**

Although using electrocardiogram (ECG) for pulmonary embolism (PE) risk stratification has shown mixed results, it is currently used as supplementary evidence in risk stratification. This cross‐sectional study aimed to assess and compare ECG findings of massive and submassive PE versus segmental PE.

**Methods:**

This cross‐sectional study included 250 hospitalized patients with a confirmed diagnosis of acute PE from 2015 to 2020 in Southern Iran. Demographic variables, clinical data, troponin levels, on‐admission ECG findings, echocardiography findings, and ECG findings 24 h after receiving anticoagulants or thrombolytics were extracted.

**Results:**

Patients diagnosed with submassive or massive PE exhibited significantly higher rates of right axis deviation (*p* = .010), abnormal ST segment (*p* < .0001), S1Q3T3 pattern (*p* < .0001), inverted T wave in leads V1–V3 (*p* < .0001), inverted T wave in leads V4–V6 (*p* < .0001), and inverted T wave in leads V1‐V6 (*p* < .0001). In a multivariable model, inverted T wave in leads V1–V3, inverted T wave in leads V4–V6, pulse rate, and positive troponin test were the statistically independent variables for predicting submassive or massive PE. Furthermore, inverted T wave in leads V1–V3 (sensitivity: 85%, specificity: 95%, accuracy: 93%, AUC: 0.902) and troponin levels (sensitivity: 72%, specificity: 86%, accuracy: 83%, AUC: 0.792) demonstrated the best diagnostic test performance for discriminating submassive or massive PE from segmental PE.

**Conclusion:**

In addition to clinical rules, ECG can serve as an ancillary tool for assessing more invasive testing and earlier aggressive treatments among patients with PE, as it can provide valuable information for the diagnosis and risk stratification of submassive or massive PE.

## Graphical abstract

Differentiating massive or submassive pulmonary embolism (PE) using electrocardiogram (ECG), can help physicians with better management. In this study 250 patients with definite diagnosis of PE were selected and ECG finding were compared. It was shown that inverted T wave in leads V1–V3 and troponin levels were the best tools.

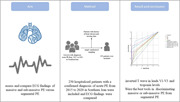

## 1 INTRODUCTION

A pulmonary embolism (PE) happens when a clot, usually formed in the leg or pelvic veins, travels to the lungs and gets stuck in a branching blood vessel. These clots can occur in different locations within the pulmonary artery, including the lobes, segments, and subsegments of the lungs.^1^

Regardless of where the clot is located, patients with shock, hypotension, end‐organ hypoperfusion, or cardiac arrest are considered high‐risk for PE. Submassive PE patients with right ventricular dysfunction^2^ and preserved systemic arterial pressure have twice the mortality rate of low‐risk PE patients, even with anticoagulation treatment. For patients with profound hypotension due to massive PE, the death rate is even higher.^3^ High‐risk PE accounts for approximately 5% of cases.^4^ In the International Cooperative Pulmonary Embolism Registry (ICOPER) study, which included 2392 patients with acute PE, the incidence of massive PE was 4.5%, with a 90‐day mortality rate of 52.4% (95% CI = 43.3, 62.1%).^5^

Electrocardiogram (ECG) abnormalities observed during PE occur due to the changes in myocardial impedance and vector of electrical currents through the heart that are caused by anatomical and functional alterations induced by the presence of PE. The most frequently encountered ECG pattern in PE is sinus tachycardia, which has limited sensitivity and specificity. Another common finding is the S1Q3T3 pattern, characterized by an S wave in lead I, a Q wave in lead III, and an inverted T wave in lead III. Other ECG abnormalities, such as right precordial inverted T wave and right bundle branch block (RBBB), are less common and also have low sensitivity and specificity.^6^ According to some reports, there is no significant correlation between ECG findings and hemodynamic compromise in non‐risk‐stratified PE patients.^7^, ^8^

However, the results regarding using ECG for PE risk stratification have been mixed. While some studies have shown that an inverted T wave in the precordial leads is predictive of massive PE,^6^ others have found that ECG patterns are abnormal in a high percentage of patients with submassive or massive PE.^9^ Additionally, a retrospective study of 48 patients with acute PE found that ECG was highly sensitive and specific (75% and 95%) when compared with biomarkers in detecting right ventricular dysfunction.^10^ Various ECG scoring systems have been developed to predict the occurrence of right ventricular overload or death. However, these scoring systems tend to be complicated. As a result, ECG is currently used as a supporting tool in risk stratification.^11^, ^12^

For our study, we aimed to evaluate and compare the ECG findings of patients diagnosed with massive and submassive PE versus those with segmental PE. The study was conducted at Namazi Hospital in Shiraz, located in the south of Iran, and covers the time period between 2015 and 2020.

## 2 MATERIALS AND METHODS

### 2.1 Study design

In this cross‐sectional study, we obtained data on patients hospitalized at Namazi Hospital, affiliated with Shiraz University of Medical Sciences, Shiraz, Iran, who were diagnosed with acute PE and admitted between 2015 and 2020. Patients were selected through simple randomized sampling. A minimum sample size of 250 was calculated based on a type I error of 5%, power of analysis of 90%, a prevalence of 60% for S1Q3T3 findings in the ECG of PE patients, and a precision of 10. Patients with a history of ischemic heart diseases, coronary artery revascularization, structural heart diseases, or missing data in the hospital health profile were excluded from the study. Informed consent was obtained from all the patients and also ethical approval was received from Shiraz University of Medical Scenes ethics committee (approval ID: IR. SUMS. MED. REC.1400.644).

### 2.2 Assessments

The study subjects were identified by using specific ICD‐10 codes for acute PE. Their health records were reviewed to extract information on demographic variables, such as age and sex, as well as clinical and laboratory data, such as chief complaint, systolic and diastolic blood pressure, pulse rate, respiratory rate, and troponin levels. In addition, their ECG findings upon admission, including axis deviation, P wave, PR interval, QRS interval, QTc, RVH, poor R progression, RBBB, left bundle branch block (LBBB), ST segment depression, ST segment elevation, inverted T wave, and S1Q3T3, as well as echocardiography findings, such as right ventricle (RV) function, McConnell sign, and tricuspid annular plane systolic excursion (TAPSE), were also recorded. Finally, ECG findings 24h after receiving anti‐coagulants or thrombolytics were also noted.

Troponin levels were measured using VIDAS analyzer which uses the same kit in all patients with cutoff point of 19 ng/L. The analysis is based on enzyme linked fluorescent assay (ELFA). Echocardiography were done using standard transthoracic technique according to American society of echocardiography guideline.^13^

### 2.3 Statistical analysis

The SPSS Statistics software (version 26.0; SPSS Statistics Inc.) was utilized to conduct analysis. The results were presented as frequency (percent) and Mean ± standard deviation. Two distinct group comparisons were conducted; the initial analysis compared massive and submassive PE with segmental PE, while the second analysis compared massive, submassive, and segmental PE. P values were reported for both sets of analyses. For qualitative variables, the Chi‐square test was employed for univariable group comparison, while the independent *t*‐test and one‐way ANOVA test were used for quantitative variables. Additionally, variables with a *p* value of less than .2 in the univariable analysis were chosen for multivariable analysis, which was conducted through logistic regression. The beta coefficient, standard error, odds ratio, 95% confidence interval (CI), and *p* value were the statistics reported for this analysis. Furthermore, ECG features were subjected to diagnostic test analysis and receiver‐operating characteristic (ROC) analysis to assess their efficacy in classifying the severity of PE. The significance level was set at a *p* value of less than .05.

## 3 RESULTS

The analysis included 250 patients diagnosed with PE, 60% of whom were male, and the mean age was 53.58 ± 19.34 years. Frothy patients (16%) had history of previous deep vein thromboembolism or pulmonary embolism. Sixty‐one patients (24.4%) had history of major surgery or fracture within last 2 months, twenty‐seven (10.8%) patients had active malignancy, seventy‐three patients (29.2%) had history of immobilization (resting in bed for more than 4 days). Hormonal therapy or oral contraceptive pills usage were only present between female with frequency of 20 patients (8% among all patients and 20% among female). Regarding these risk factors 124 patients had no risk factors based on our history, and 126 patients had at least one risk factor. Among these patients, 189 (75.6%), 48 (19.2%), and 13 (5.2%) had segmental, submassive, and massive PE, respectively.

Patients with submassive or massive PE were predominantly female (P first, second = 0.011, 0.006) and older (P first, second = 0.001, 0.033) and exhibited significantly higher pulse rates (P first, second = 0.003, 0.019), positive troponin test results (P first, second < 0.0001), and dysfunctional right ventricles (P first, second < 0.0001), as well as the McConnell sign (P first, second < 0.0001). Moreover, they had a lower mean TAPSE compared to those with segmental PE (P first, second < 0.0001). Additionally, massive PE was linked with significantly lower mean arterial systolic and diastolic blood pressures than segmental and submassive PE (*p* < .0001 and 0.001, respectively). However, this difference disappeared when comparing submassive or massive PE with segmental PE (*p* = .717 and .251, respectively). The groups did not differ significantly with respect to the chief complaint (P first, second = 0.278, 0.200) and respiratory rate (P first, second = 0.195, 0.297) (refer to Table 1 and Supplementary Table 1).

**Table 1:** Comparison of demographic, clinical, laboratory, and echocardiographic characteristics of patients with submassive or massive PE with those of patients with segmental PE.

Variable	Total (250)	Segmental (*n* = 189)	Submassive or massive (*n* = 61)	*p* a
**Sex**				
***Male***	**150 (60.0)** ^b^	**122 (64.6)**	**28 (45.9)**	**.011**
***Female***	**100 (40.0)**	**67 (35.4)**	**33 (54.1)**	
**Age**	**53.58** ± **19.34** ^c^	**51.83** ± **19.53**	**59.00** ± **17.81**	**.011** d
Chief complaint				
*Dyspnea*	**201 (80.4)**	156 (82.5)	45 (73.8)	.278
*Chest pain*	**24 (9.6)**	17 (9.0)	7 (11.5)	
*Other*	**25 (10.0)**	16 (8.5)	9 (14.8)	
SBP	119.78 ± 18.21	120.04 ± 17.15	118.95 ± 21.28	.717^d^
DBP	74.87 ± 11.14	75.33 ± 10.76	73.44 ± 12.23	.251^d^
**Pulse rate**	**99.58** ± **17.49**	**97.84** ± **17.91**	**104.97** ± **15.03**	**.003** ^d^
Respiratory rate	20.39 ± 3.44	20.20 ± 3.07	20.98 ± 4.37	.195^d^
**Troponin**				
***Positive***	**70 (28.0)**	**26 (13.8)**	**44 (72.1)**	<**.0001**
***Negative***	**180 (72.0)**	**163 (86.2)**	**17 (27.9)**	
RV function				
*Normal*	194 (77.6)	188 (99.5)	6 (9.8)	<.0001^e^
*Dysfunctional*	56 (22.4)	1 (0.5)	55 (90.2)	
McConnell sign				
*Positive*	20 (8.0)	0 (0)	20 (32.8)	<.0001^e^
*Negative*	230 (92.0)	189 (100)	41 (67.2)	
TAPSE	19.94 ± 3.47	21.21 ± 2.46	16.02 ± 3.20	<.0001^d^

*Note*: Bold variables represent that they selected for the multivariable analysis.

Abbreviations: DBP, diastolic blood pressure; RV, right ventricle; SBP, systolic blood pressure; TAPSE, tricuspid annular plane systolic excursion.

^a^ Chi‐square test.

^b^ Frequency (percent).

^c^ Mean ± standard deviation.

^d^ P independent *t*‐test.

^e^ Underpowered analysis.

The rates of right axis deviation, abnormal ST segment, S1Q3T3 pattern, inverted T wave in lead V1–V3, inverted T wave in leads V4–V6, and global inverted T wave were significantly higher in patients with submassive or massive PE, according to two different analysis sets (P first, second = 0.010, 0.013; P first, second < 0.0001; P first, second < 0.0001; P first, second < 0.0001; P first, second < 0.0001; P first, second < 0.0001, respectively). Massive PE also had a significantly higher rate of left axis deviation compared to segmental and submassive PE (*p* = .009), but this difference disappeared when comparing submassive or massive PE with segmental PE (*p* = .525). However, there were no statistically significant differences between groups with respect to right ventricular hypertrophy, poor R progression, left and right bundle branch blocks, QRS fragmentation, and QTc prolongation (see Table 2 and Supplementary table 2).

**Table 2:** Comparison of electrocardiographic findings between patients with submassive or massive PE and patients with segmental PE.

Finding	Total (250)	Segmental (*n* = 189)	Submassive or massive (*n* = 61)	*p* a
**Right axis deviation**				
***Negative***	**243 (97.2)** b	**187 (98.9)**	**56 (91.8)**	**.010** ^c^
***Positive***	**7 (2.8)**	**2 (1.1)**	**5 (8.2)**	
Left axis deviation				
*Negative*	237 (94.8)	180 (95.2)	57 (93.4)	.525
*Positive*	13 (5.2)	9 (4.8)	4 (6.6)	
Right ventricular hypertrophy				
*Negative*	242 (96.8)	184 (97.4)	58 (95.1)	.408^c^
*Positive*	8 (3.2)	5 (2.6)	3 (4.9)	
Poor R progression				
*Negative*	243 (97.2)	184 (97.4)	59 (96.7)	.680^c^
*Positive*	7 (2.8)	5 (2.6)	2 (3.3)	
**Right bundle branch block**				
***Negative***	**242 (96.8)**	**185 (97.9)**	**57 (93.4)**	**.102** ^c^
***Positive***	**8 (3.2)**	**4 (2.1)**	**4 (6.6)**	
Left bundle branch block				
*Negative*	244 (97.6)	184 (97.4)	60 (98.4)	1.000^c^
*Positive*	6 (2.4)	5 (2.6)	1 (1.6)	
P wave				
*Normal*	250 (100)	189 (100)	61 (100)	
*Abnormal*	0 (0)	0 (0)	0 (0)	
PR interval				
*Normal*	250 (100)	189 (100)	61 (100)	—
*Abnormal*	0 (0)	0 (0)	0 (0)	
ST segment				
*Normal*	218 (87.2)	187 (98.9)	31 (50.8)	<.0001^c^
*Elevation* d	11 (4.4)	1 (0.5)	10 (16.4)	
*Depression* e	21 (8.4)	1 (0.5)	20 (32.8)	
**ST segment**				
***Normal***	**218 (87.2)**	**187 (98.9)**	**31 (50.8)**	<**.0001** c
***Abnormal***	**32 (12.8)**	**2 (1.1)**	**30 (49.2)**	
QRS fragmentation				
*Negative*	246 (98.4)	187 (98.9)	59 (96.7)	.251^c^
*Positive*	4 (1.6)	2 (1.1)	2 (3.3)	
Deep S wave in lead I (S1)				
*Negative*	105 (82.0)	168 (88.9)	37 (60.7)	<.0001
*Positive*	45 (18.0)	21 (11.1)	24 (39.3)	
Pathologic Q wave in lead III (Q3)				
*Negative*	187 (74.8)	152 (80.4)	35 (57.4)	.001
*Positive*	63 (25.2)	37 (19.6)	26 (42.6)	
Inverted T wave in lead III (T3)				
*Negative*	223 (89.2)	185 (97.9)	38 (62.3)	<.0001^c^
*Positive*	27 (10.8)	4 (2.1)	23 (37.7)	
**S1Q3T3 pattern**				
***Negative***	**231 (92.4)**	**187 (98.9)**	**44 (72.1)**	<**.0001**
***Positive***	**19 (7.6)**	**2 (1.1)**	**17 (27.9)**	
**Inverted T wave in lead V1–V3**				
***Negative***	**189 (75.6)**	**180 (95.2)**	**9 (14.8)**	<**.0001** c
***Positive***	**61 (24.4)**	**9 (4.8)**	**52 (85.2)**	
**Inverted T wave in leads V4–V6**				
***Negative***	**213 (85.2)**	**186 (98.4)**	**27 (44.3)**	<**.0001** c
***Positive***	**37 (14.8)**	**3 (1.6)**	**34 (55.7)**	
Inverted T wave in leads V1–V6 (global)				
*Negative*	242 (96.8)	189 (100)	53 (86.9)	<.0001^c^
*Positive*	8 (3.2)	0 (0)	8 (13.1)	
QTc prolongation				
*Negative*	248 (99.2)	187 (98.9)	61 (100)	1.000^c^
*Positive*	2 (0.8)	2 (1.1)	0 (0)	

*Note*: Bold variables represent that they selected for the multivariable analysis.

^a^ Chi‐square test.

^b^ Frequency (percent).

^c^ Underpowered analysis.

^d^ STe at V1–V3 (5 [2.0%]), STe at V1–V4 (4 [1.6%]), STe at V1–V5 (1 [0.4%]), STe at V2–V5 (1 [0.4%]).

^e^ STd at V1–V3 (7 [2.8%]), STd at V1–V4 (4 [1.6%]), STd at V1–V5 (5 [2.0%]), STd at V1–V6 (3 [1.2%]), STd at V2–V6 (1 [0.4%]), STd at V5–V6 (1 [0.4%]).

The multivariable model selected 10 demographic, clinical, and electrocardiographic variables, and the analysis identified inverted T wave in leads V1–V3 (*p* < .0001), inverted T wave in leads V4–V6 (*p* = .024), pulse rate (*p* = .015), and positive troponin test (*p* = .018) as independent variables for predicting submassive or massive PE. The odds ratios for these variables were 33.224 (95% CI = 7.725, 142.893), 8.171 (95% CI = 1.316, 50.751), 1.052 (95% CI = 1.010, 1.096), and 4.131 (95% CI = 1.270, 13.439), respectively. The model accounted for 77.9% of the variance based on the Nagelkerke R Square statistic (see Table 3).

**Table 3:** Multivariable analysis on the selected demographic, clinical and electrocardiographic variables for identifying the significant independent predictors of submassive or massive PE.

Variable	B	SE	OR	95% CI	*p*
Age	0.006	0.016	1.006	0.976, 1.038	.696
Male sex	0.357	0.614	1.429	0.429, 4.763	.561
**Pulse rate**	**0.051**	**0.021**	**1.052**	**1.010, 1.096**	**.015**
**Positive troponin test**	**1.418**	**0.602**	**4.131**	**1.270, 13.439**	**.018**
Right axis deviation	1.969	1.295	7.164	0.566, 90.649	.128
Right bundle branch block	0.892	1.661	2.440	0.094, 63.234	.591
Abnormal ST segment	1.309	0.994	3.702	0.528, 25.971	.188
S1Q3T3 pattern	−0.558	1.096	0.572	0.067, 14.900	.610
**Inverted T wave in leads V1–V3**	**3.503**	**0.744**	**33.224**	**7.725, 142.893**	**<.0001**
**Inverted T wave in leads V4–V6**	**2.101**	**0.932**	**8.171**	**1.316, 50.751**	**.024**
Intercept	−9.497	2.567	—	—	—

*Note*: Model accuracy: 94.0%. Bold variables represent the significant independent predictive variables.

Abbreviations: SE, standard error; OR, odds ratio, CI, confidence interval.

**Figure 1:**
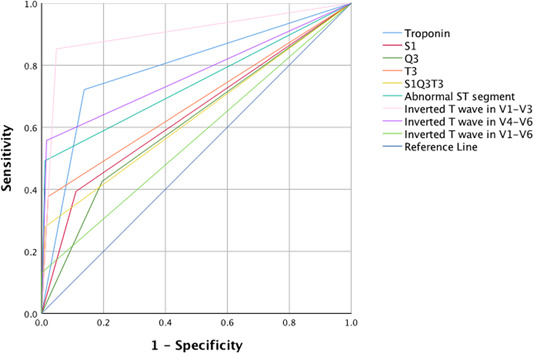
ROC plot of ECG findings for discriminating massive and submassive PE from segmental PE.

The best diagnostic test performance for distinguishing submassive or massive PE from segmental PE was achieved by inverted T wave in leads V1–V3 (with a sensitivity of 85%, specificity of 95%, accuracy of 93%, and an area under curve (AUC) of 0.902) and troponin levels (with a sensitivity of 72%, specificity of 86%, accuracy of 83%, and an AUC of 0.792) (see Table 4 and Figure 1).

**Table 4:** Diagnostic test performance of ECG findings for discriminating between massive and submassive PE and segmental PE.

Variable	Sensitivity	Specificity	PPV	NPV	Accuracy	AUC	SE	Lower	Upper
Troponin	0.72	0.86	0.63	0.91	0.83	0.792	0.037	0.720	0.864
S1	0.39	0.89	0.53	0.82	0.77	0.641	0.044	0.555	0.727
Q3	0.43	0.80	0.41	0.81	0.71	0.615	0.043	0.530	0.700
T3	0.38	0.98	0.85	0.83	0.83	0.678	0.045	0.591	0.765
S1Q3T3	0.28	0.99	0.90	0.81	0.82	0.634	0.45	0.546	0.723
Abnormal ST segment	0.49	0.99	0.94	0.86	0.87	0.741	0.43	0.657	0.825
Inverted T/V1–V3	0.85	0.95	0.85	0.95	0.93	0.902	0.028	0.847	0.957
Inverted T/V4–V6	0.56	0.98	0.92	0.87	0.88	0.771	0.041	0.690	0.852
Inverted T/global	0.13	1.00	1.00	0.78	0.79	0.566	0.045	0.478	0.653

Abbreviations: AUC, area under curve; NPV, negative predictive value; PPV, positive predictive value; SE, standard error.

## 4 DISCUSSION

We conducted a retrospective study involving 250 patients diagnosed with PE, revealing a prevalence of 24.4% for submassive and massive PE (5.12% and 19.2%, respectively). The study analyzed the patients' clinical, echocardiographic, laboratory, and ECG findings. The results showed that patients with submassive or massive PE had significantly higher rates of various ECG abnormalities, including right axis deviation, abnormal ST segment, S1Q3T3 pattern, inverted T wave in lead V1–V3, inverted T wave in leads V4–V6, and global inverted T wave (leads V1–V6). Furthermore, the study identified the statistically independent variables for predicting submassive or massive PE, including inverted T wave in leads V1–V3, inverted T wave in leads V4–V6, pulse rate, and positive troponin test. The study found that these variables were useful in predicting the odds of submassive or massive PE. Finally, the study also evaluated the diagnostic test performance of ECG findings for discriminating submassive or massive PE from segmental PE. The results indicated that inverted T wave in leads V1–V3 and troponin levels showed the best diagnostic test performance for discriminating submassive or massive PE from segmental PE.

In general, different ECG characteristics in massive and submassive PE may result from the strain on the right ventricle.^14^ The ECG pattern of right ventricle strain is known to be linked with the severity and unfavorable short‐term outcome, which can provide additional prognostic information to echocardiography findings of right ventricle function in patients with acute PE.^15^

Our study revealed that nearly half (49.2%) of the patients with massive or submassive PE had abnormal ST segments, with 32.8% exhibiting ST depression and 16.4% displaying ST elevation. In contrast, only 1.1% of patients with segmental PE exhibited ST segment abnormalities. Previous research has shown that ST segment depression in leads V4–V6^12^ or in leads I, II, and V4–V6^16^ is significantly more common in PE patients who died compared to those who survived, as well as in patients who experienced in‐hospital complications compared to those who did not. Furthermore, ST depression in leads V4‐V6,^12^, ^14^, ^17^ I, II and V4–V6,^16^ and V1–V3/V4^18^ has been linked to poor outcomes such as cardiogenic shock, positive troponin levels, hemodynamic instability, and mortality. Additionally, Omar^18^ reported that ST segment elevation in leads V1–V3/V4 indicates intermediate to high‐risk PE patients. These findings suggest that ST segment abnormalities may be useful in assessing the severity and prognosis of PE.

It can be difficult to distinguish between massive or submassive PE and anterior/inferior ST elevation myocardial infarction (STEMI) because of concerns about the fatal consequences of STEMI. Therefore, clinicians should keep in mind the possibility of PE in patients with a history and clinical presentation that suggest it and prompt earlier performance of computed tomography pulmonary angiography (CTPA) if ST‐segment elevation is present.^8^, ^19^ It is worth noting that some authors have suggested that simultaneous inverted T waves in anterior and inferior leads can be an important clue favoring massive and submassive PE.^19^ In these cases, right ventricle dilation toward the left leads to the movement of inverted T waves toward the left side. A study showed that inverted T waves in leads III (which faces the inferior region of the right ventricle) and V1–2 (which faces the anterior region of the right ventricle), as well as the maximum magnitude of inverted T waves in leads V1–2, identified PE with 98% sensitivity and 92% specificity.^19^

Our study found that a large proportion of patients with massive or submassive PE had inverted T waves in various leads, with significantly lower rates observed in patients with segmental PE. Specifically, 37.7%, 85.2%, 55.7%, and 13.1% of patients with massive or submassive PE had inverted T waves in lead III, V1–V3, V4–V6, and V1–V6 (global), respectively. Inverted T waves in leads V1–V3 and V4–V6 were found to be statistically independent variables for predicting massive or submassive PE. Additionally, inverted T waves in leads V1–V3 had the highest diagnostic performance for discriminating between submassive or massive PE and segmental PE. Previous research has shown that inverted T waves in precordial leads are a common and early finding in confirmed cases of massive PE.^14^ In line with our findings, a study conducted by Ferrari et al.^8^ revealed that inverted T wave in leads V1–V3 was the most prevalent and dependable indicator of massive acute PE. Another investigation found a 70% incidence rate of inverted T wave in leads V1–V3/V4 among patients with pulmonary artery trunk or main pulmonary artery embolism, with a significantly higher occurrence of inverted T waves in these patients compared to those with lobar artery or remote branch embolism. Additionally, another study indicated that in more severe PE patients with right ventricular dysfunction, inverted T wave in leads V1–V3 had higher sensitivity and overall diagnostic accuracy compared to the classical S1Q3T3 pattern and right bundle branch block.^14^

Our study also revealed that there was an 8.2% prevalence of right axis deviation in patients with massive or submassive PE, which was significantly higher than the 1.1% prevalence in patients with segmental PE. Similarly, a study by Ermis et al.^20^ found a higher incidence of right axis deviation in patients with massive (28%) and submassive (15%) PE when compared to those with low‐risk PE (3%).

We observed a significantly higher prevalence of positive troponin test among patients with sub massive or massive PE compared to those with segmental PE. Troponin level was also identified as one of the best diagnostic test performances for distinguishing sub massive or massive PE from segmental PE. Consistent with previous research, our findings indicate that troponin levels are closely linked to right ventricular overload and dilatation in PE, particularly in cases of massive and sub massive PE.^9^, ^12^, ^21^ In patients with massive or submassive PE, the influence of the embolism on the hemodynamics of the right heart is greater, resulting in a greater rise in the level of myocardial troponin compared to those with remote branch embolism.^22^ Elevated troponin levels are associated with prolonged hypotension, cardiogenic shock, and the need for resuscitation, mechanical ventilation, and inotropic support.^23^ Moreover, troponin levels may be normal in patients with low‐risk PE.^24^

## 5 CONCLUSION

Our study identified a number of ECG indicators that can distinguish between massive/submassive PE and segmental PE. These findings suggest that ECG can be used in conjunction with clinical assessment tools, such as the Geneva score, to help determine the need for invasive testing and more aggressive treatments, such as thrombolysis and heparin infusion, in patients with PE. Specifically, we found that inverted T waves in leads V1–V3 had the highest overall diagnostic accuracy for identifying massive/submassive acute PE, compared to the classical ECG findings.

## AUTHOR CONTRIBUTIONS

All authors were involved in conceiving and designing the study. Data collection was conducted by Zahra Bahreini and Maliheh Kamali, while Mehdi Bazrafshan performed data analysis. Zahra Bahreini, Shahrokh Sadeghi boogar, and Fatemeh Kheshty drafted the manuscript, which was critically revised by Hamed Bazrafshan drissi. Mehdi Bazrafshan supervised the manuscript preparation.

## CONFLICT OF INTEREST STATEMENT

The authors declare that they have no competing interests that are relevant to this specific article.

## Supporting information

Supporting Information.

## Data Availability

The data that support the findings of this study are available on request from the corresponding author. The data are not publicly available due to privacy or ethical restrictions.
