# Supplementary material for: Differentiating electrocardiographic indications of massive and submassive pulmonary embolism: A cross‐sectional study in Southern Iran from 2015 to 2020
Source: Clin Cardiol. 2024 Mar 11;47(3):e24252. doi: 10.1002/clc.24252 (PMC10926280; doi:10.1002/clc.24252)
Supplement: Supplementary file 1 — Supporting Information. [file CLC-47-e24252-s001.docx]

| **Supplementary table 1.** Comparison of demographic, clinical, laboratory, and echocardiographic characteristics of patients with segmental, sub massive, and massive PE | | | | |
| --- | --- | --- | --- | --- |
| Variable | Segmental  (n = 189) | Sub massive  (n = 48) | Massive  (n = 13) | P ^2^ |
| Sex  *Male*  *Female* | 122 (64.6) ^1^  67 (35.4) | 25 (52.1)  23 (47.9) | 3 (23.1)  10 (76.9) | 0.006 ^†^ |
| Age | 51.83 ± 19.53 ^3^ | 59.83 ± 17.81 | 55.92 ± 18.16 | 0.033 ^4^ |
| Chief complaint  *Dyspnea*  *Chest pain*  *Other* | 156 (82.5)  17 (9.0)  16 (8.5) | 35 (72.9)  7 (14.6)  6 (12.5) | 10 (76.9)  0 (0)  3 (23.1) | 0.200 ^†^ |
| SBP | 120.04 ± 17.15 | 123.73 ± 20.42 | 101.31 ± 14.25 | <0.0001 ^4^ |
| DBP | 75.33 ± 10.76 | 76.15 ± 11.91 | 63.46 ± 7.47 | 0.001 ^4^ |
| Pulse rate | 97.84 ± 17.91 | 105.50 ± 15.85 | 103.00 ± 11.85 | 0.019 ^4^ |
| Respiratory rate | 20.20 ± 3.07 | 20.96 ± 4.67 | 21.08 ± 3.20 | 0.297 ^4^ |
| Troponin  *Positive*  *Negative* | 26 (13.8)  163 (86.2) | 32 (66.7)  16 (33.3) | 12 (92.3)  1 (7.7) | <0.0001 ^†^ |
| RV function  *Normal*  *Dysfunctional* | 188 (99.5)  1 (0.5) | 6 (12.5)  42 (87.5) | 0 (0)  13 (100) | <0.0001 ^†^ |
| McConnell sign  *Positive*  *Negative* | 0 (0)  189 (100) | 8 (16.7)  40 (83.3) | 12 (92.3)  1 (7.7) | <0.0001 ^†^ |
| TAPSE | 21.21 ± 2.46 | 16.79 ± 2.97 | 13.15 ± 2.30 | <0.0001 ^4^ |
| ^1^ Frequency (percent)  ^2^ Chi-square test  ^3^ Mean ± standard deviation  ^4^ P _One-way ANOVA_  Abbreviations: RV, right ventricle; TAPSE, tricuspid annular plane systolic excursion; SBP, systolic blood pressure; DBP, diastolic blood pressure.  Bold variables represent that they selected for the multivariable analysis.  ^†^ Underpowered analysis | | | | |

| **Supplementary table 2.** Comparison of electrocardiographic findings of patients with segmental, sub massive, and massive PE | | | | |
| --- | --- | --- | --- | --- |
| Finding | Segmental  (n = 189) | Sub massive  (n = 48) | Massive  (n = 13) | P ^2^ |
| Right axis deviation  *Negative*  *Positive* | 187 (98.9) ^1^  2 (1.1) | 44 (91.7)  4 (8.3) | 12 (92.3)  1 (7.7) | 0.013 ^†^ |
| Left axis deviation  *Negative*  *Positive* | 180 (95.2)  9 (4.8) | 47 (97.9)  1 (2.1) | 10 (76.9)  3 (23.1) | 0.009 ^†^ |
| Right ventricular hypertrophy  *Negative*  *Positive* | 184 (97.4)  5 (2.6) | 46 (95.8)  2 (4.2) | 12 (92.3)  1 (7.7) | 0.555 ^†^ |
| Poor R progression  *Negative*  *Positive* | 184 (97.4)  5 (2.6) | 46 (95.8)  2 (4.2) | 13 (100)  0 (0) | 0.698 ^†^ |
| Right bundle branch block  *Negative*  *Positive* | 185 (97.9)  4 (2.1) | 44 (91.7)  4 (8.3) | 13 (100)  0 (0) | 0.073 ^†^ |
| Left bundle branch block  *Negative*  *Positive* | 184 (97.4)  5 (2.6) | 47 (97.9)  1 (2.1) | 13 (100)  0 (0) | 0.823 ^†^ |
| P wave  *Normal*  *Abnormal* | 189 (100)  0 (0) | 48 (100)  0 (0) | 13 (100)  0 (0) | - |
| PR interval  *Normal*  *Abnormal* | 189 (100)  0 (0) | 48 (100)  0 (0) | 13 (100)  0 (0) | - |
| ST segment  *Normal*  *Elevation*  *Depression* | 187 (98.9)  1 (0.5)  1 (0.5) | 27 (56.3)  7 (14.6)  14 (29.2) | 4 (30.8)  3 (23.1)  6 (46.2) | <0.0001 ^†^ |
| ST segment  *Normal*  *Abnormal* | 187 (98.9)  2 (1.1) | 27 (56.3)  21 (43.8) | 4 (30.8)  9 (69.2) | <0.0001 ^†^ |
| QRS fragmentation  *Negative*  *Positive* | 187 (98.9)  2 (1.1) | 46 (95.8)  2 (4.2) | 13 (100)  0 (0) | 0.276 ^†^ |
| Deep S wave in lead I [S1]  *Negative*  *Positive* | 168 (88.9)  21 (11.1) | 31 (64.6)  17 (35.4) | 6 (46.2)  7 (53.8) | <0.0001 |
| Pathologic Q wave in lead III [Q3]  *Negative*  *Positive* | 152 (80.4)  37 (19.6) | 29 (60.4)  19 (39.6) | 6 (46.2)  7 (53.8) | 0.001 |
| Inverted T wave in lead III [T3]  *Negative*  *Positive* | 185 (97.9)  4 (2.1) | 31 (64.6)  17 (35.4) | 7 (53.8)  6 (46.2) | <0.0001 ^†^ |
| S1Q3T3 pattern  *Negative*  *Positive* | 187 (98.9)  2 (1.1) | 37 (77.1)  11 (29.1) | 7 (53.8)  6 (46.2) | <0.0001 ^†^ |
| Inverted T wave in lead V1-V3  *Negative*  *Positive* | 180 (95.2)  9 (4.8) | 8 (16.7)  40 (83.3) | 1 (7.7)  12 (92.3) | <0.0001 ^†^ |
| Inverted T wave in leads V4-V6  *Negative*  *Positive* | 186 (98.4)  3 (1.6) | 20 (41.7)  28 (58.3) | 7 (53.8)  6 (46.2) | <0.0001 ^†^ |
| Inverted T wave in leads V1-V6 (global)  *Negative*  *Positive* | 189 (100)  0 (0) | 41 (85.4)  7 (14.6) | 12 (92.3)  1 (7.7) | <0.0001 ^†^ |
| QTc prolongation  *Negative*  *Positive* | 248 (99.2)  2 (0.8) | 187 (98.9)  2 (1.1) | 61 (100)  0 (0) | 1.000 ^†^ |
| ^1^ Frequency (percent)  ^2^ Chi-square test  ^†^ Underpowered analysis | | | | |
